# Farm use of calcium hydroxide as an effective barrier against pathogens

**DOI:** 10.1038/s41598-021-86796-w

**Published:** 2021-04-12

**Authors:** Shinji Matsuzaki, Kento Azuma, Xuguang Lin, Masahiro Kuragano, Koji Uwai, Shinya Yamanaka, Kiyotaka Tokuraku

**Affiliations:** Department of Applied Science, Muroran Institute of Technology, Muroran, Hokkaido Japan

**Keywords:** Antimicrobials, Policy and public health in microbiology

## Abstract

Livestock farming is affected by the occurrence of infectious diseases, but outbreaks can be prevented by proper sanitary control measures. Calcium hydroxide (Ca(OH)_2_), commonly called slaked lime, powder is traditionally used as a disinfectant to prevent infectious diseases in livestock. Since Ca(OH)_2_ can inactivate a wide variety of pathogens, has a small environmental impact, does not require a disinfection tank (i.e., can be spread directly on the ground) and is produced inexpensively worldwide, it is used for the prevention of epidemics on farms worldwide. Water is essential for the strong alkalinity that underlies its disinfecting effect, but it is unknown how much water is required under field conditions. In addition, Ca(OH)_2_ reacts with carbon dioxide in the environment, reducing its pH, but it is unclear how long its degradation takes under actual field use. Thus, we measured the water adsorption ability of Ca(OH)_2_-based disinfectants and its relation to disinfectant activity, as assessed by colony counts and live/dead staining and observation. We found that 15–20% (w/w) water in Ca(OH)_2_ was necessary for disinfection to occur in practice. Moreover, we found that the pH of Ca(OH)_2_ decreased within about two weeks to one month under actual use in practical conditions and lost its ability to disinfect. We further showed that granules prepared from Ca(OH)_2_ and zeolite maintained high alkalinity more than twice as long as calcium powder. These findings will help to establish a suitable method of applying Ca(OH)_2_ to protect farms from infectious diseases.

## Introduction

**Figure 1:**
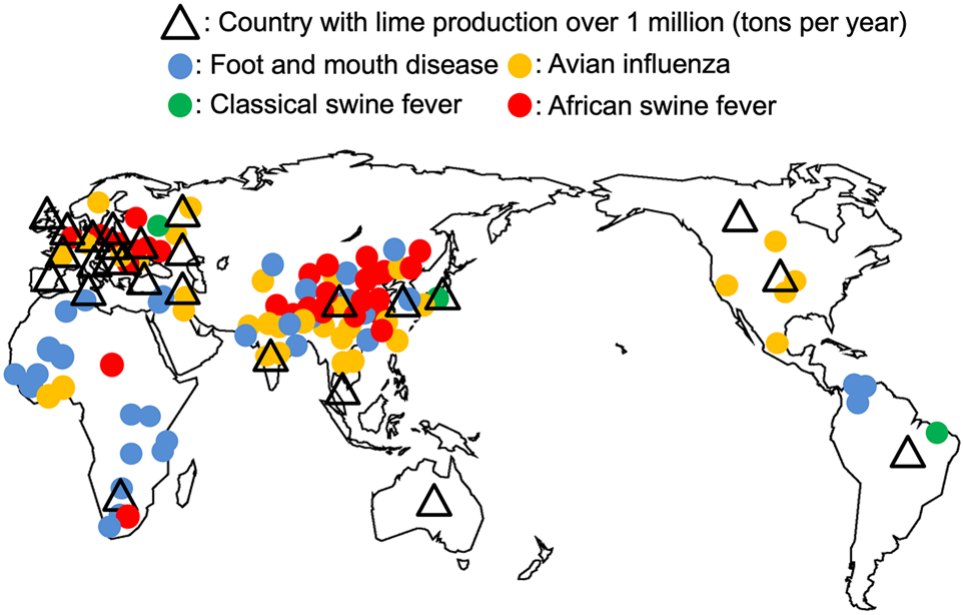
Locations of lime production and outbreaks of infectious diseases in domestic animals. Countries with lime production over 1 million tons per year, and locations of outbreaks of foot-and-mouth disease, avian influenza, classical swine fever and African swine fever in 2018–2019 (lime production was quoted from Mineral Commodity Summaries 2019^11^, and the location of livestock infectious diseases was quoted from the World Animal Health Information System Interface^2^, and we drew the world map summarizing them).

Livestock infectious diseases are prevalent worldwide (Fig. 1). For example, recently, the global impact of African swine fever (ASF; Fig. 1, red circles) in 2018 was severe, as 6.8 million pigs were killed, and since 900 million livestock pigs were raised that year, 0.75% were killed by ASF alone^1,2^. The destruction of the environment caused by an increase in livestock is regarded as a critical management issue^3,4^, so farms need to be protected against livestock infectious diseases to prevent the unnecessary death of livestock. Since many farms exist in regions with unfavorable economic conditions, disease prevention using inexpensive and universal disinfectant systems is needed to increase livestock food supply production.

It is well known that strong alkalinity inactivates various pathogens such as bacteria and viruses. After a foot-and-mouth (FMD) disease outbreak occurred in 2010, with 290,000 livestock killed in Miyazaki, Japan^5^, the Japanese Ministry of Agriculture, Forestry, and Fisheries strongly recommended the use of Ca(OH)_2_ to prevent livestock infectious diseases. In this so-called “stand-by disinfection” system, Ca(OH)_2_ powder is spread at the entrances to a farm and around livestock houses, or related facilities as a livestock hygiene service center at a density of approximately 0.5–1.0 kg/m^2^ (Supplementary Fig. 1). The strong alkalinity (pH 12.4, 25 °C) of Ca(OH)_2_ prevents the invasion of pathogens brought to the premises by cars, people, wild animals, etc., and acts as a barrier against various infectious diseases by inactivating bacteria and viruses. *Escherichia coli*, *Salmonella*, and highly pathogenic avian influenza are inactivated in 24 h, 2 h, and 5–10 min, respectively^6–8^.

Disinfection by Ca(OH)_2_ is related to the release of hydroxyl ions in an aqueous environment^6,8,9^. However, no studies have examined the disinfectant properties of solid Ca(OH)_2_ under stand-by disinfection conditions, that is, when a particulate-bound pathogen is placed into contact with Ca(OH)_2_ powder, which occurs during field application. Rather, the standard protocols for evaluating disinfectants, as stipulated by the European Norm (EN), the American Society for Testing and Materials (ASTM), and the Association of Official Analytical Chemists (AOAC), consist of dropping disinfectant solutions on agar or mixing a solution with liquid medium.

Ca(OH)_2_ costs 109 US dollars/ton, which is approximately one twentieth of the cost of using a surfactant as a disinfectant (2066 dollars/ton)^10^. In addition, Ca(OH)_2_ can be spread directly on the ground, i.e. no water tank is required. Since Ca(OH)_2_ does not significantly affect soil microflora^11^ because it reacts with CO_2_ in the environment to form nontoxic calcium carbonate (CaCO_3_), it is an environmentally friendly disinfectant. Furthermore, since limestone, which is the raw material for Ca(OH)_2_ production, is produced in large quantities worldwide^12^ (Fig. 1, open triangles), Ca(OH)_2_ can be widely used on farms.

In spite of its many strengths, there are several disadvantages of using Ca(OH)_2_ as a disinfectant: (1) Ca(OH)_2_ powder is scattered by wind, (2) the amount of water required for stand-by disinfection conditions is unknown, and (3) Ca(OH)_2_ and its environmental transformation product CaCO_3_, which lacks disinfection activity, are indistinguishable using common methods. By overcoming these disadvantages, realistic and effective barriers can be built to protect farms around the world against the threat of infectious diseases.

To the best of our knowledge, this report is the first to examine the effect of water content the disinfection ability of Ca(OH)_2_. We used a stand-by disinfection strategy that considers those conditions under which Ca(OH)_2_ has the highest disinfection activity. The disinfection effects of Ca(OH)_2_ (slaked lime) powder and newly developed slaked lime-based granules (SLBGs)^13^ were evaluated. SLBGs were developed to prevent scattering by wind and zeolite was added as an expected deodorant effect and for the long-term persistence of pH. These evaluations were made under conditions mimicking actual stand-by disinfection conditions, while appropriate water content was also examined.

## Results and discussion

### Preparation of SLBGs

SLBGs, which scatter in the wind less readily than powdered Ca(OH)_2_, were made from Ca(OH)_2_ powder and natural zeolite powder with a high shear wet granulator without any binder, according to our recent report^13^. In this study, sieved granules of 1.0 to 2.0 mm were used for the tests.

### pH changes persist for up to 1 month following Ca(OH)_2_ application by stand-by disinfection

**Figure 2:**
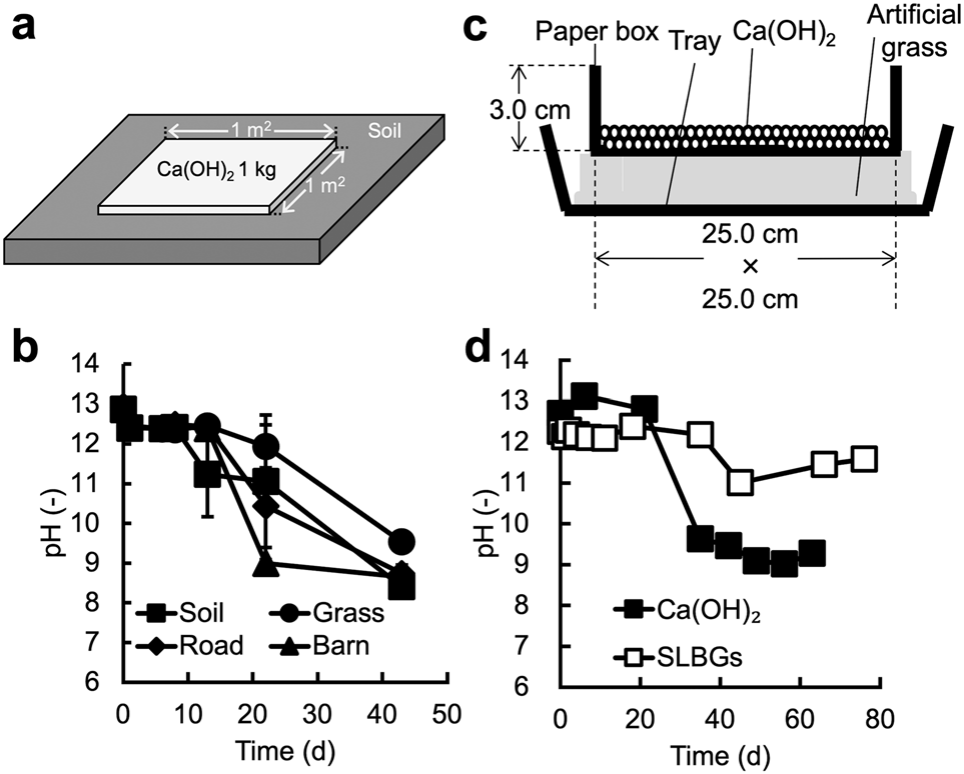
Evaluation of pH persistence under stand-by disinfection conditions. (**a**) Typical outdoor test. Ca(OH)_2_ powder was spread at 1 kg/m^2^. (**b**) In the outdoor test, the change in pH after treatment with Ca(OH)_2_ powder was evaluated on soil, grass, a road, and in front of a barn. (**c**) Schematic of the indoor test to measure the pH following disinfectant treatment. To mimic the outdoor conditions, this system consisted of a tray, artificial grass, and a paper box. For the treatments, disinfectants were placed in a paper box. Water (200 cm^3^/day) was applied, equal the annual precipitation in Hokkaido prefecture, Japan. (**d**) In the indoor test, the pH after treatment with Ca(OH)_2_ powder or SLBGs was evaluated. Data represent the mean from three separate experiments. Error bars show the standard deviation.

To date, there have been no reports examining how long the high pH underlying disinfection effects is maintained under stand-by disinfection conditions. First, we evaluated the persistence of pH changes occurring in Ca(OH)_2_ powder applied by stand-by disinfection (Fig. 2a) to soil, grass, a road, and in front of a barn (Supplementary Fig. 2). Farm vehicles and people pass frequently on a road and in front of a barn, but not on soil and grass. In front of a barn, livestock pass, in addition to vehicles and people. In this test (Fig. 2b), the high alkalinity of Ca(OH)_2_-treated surfaces began to decrease after approximately two weeks. After 44 days, their pH had decreased to approximately 9, which is comparable to the pH of CaCO_3_. In front of the barn, the pH dropped to 8.99 after 22 days (Fig. 2b, closed triangles). These results suggest that the disinfection effect resulting from the high alkalinity of Ca(OH)_2_ lasted only approximately 2 weeks to 1 month under stand-by disinfection conditions. In another study, the pH and disinfection effect of Ca(OH)_2_ powder decreased after alternating wet and dry conditions^14^, suggesting that the decrease in pH during the one month was due to environmental conditions. The fastest drop in pH in front of the barn may have been due to Ca(OH)_2_ contact with livestock manure, as the CO_2_ generated by heterotrophic microorganisms in manure can neutralize hydroxyl ions^15^.

In the outdoor test, the results varied depending on the conditions at the test location, for example, rainfall fluctuation, the frequency of passing vehicles, and contact with manure. To determine the persistence of pH changes under constant conditions, we carried out an indoor test with conditions similar to those in stand-by disinfection (Fig. 2c). In the indoor test, the pH of the Ca(OH)_2_ powder remained high through day 21, but decreased to 9.63 on day 35 (Fig. 2d, black squares). Since this result was similar to that observed in the outdoor test (Fig. 2b), the indoor test was considered to represent the conditions of the actual outdoor stand-by disinfection. The pH of SLBGs after 76 days was 11.58 (Fig. 2d, white squares), suggesting that the disinfecting effect of SLBGs was maintained for more than twice longer than Ca(OH)_2_ powder. These results suggest that the Ca(OH)_2_ powder could maintain high alkalinity for only up to about one month under the stand-by disinfection conditions tested in this study while the SLBGs we developed maintained high alkalinity for at least twice as long. In the process of preparing SLBGs, we used water and dried it at 60 °C overnight (see methods section). During this process, Ca(OH)_2_ in SLBGs is expected to be partially carbonated. Nevertheless, SLBGs maintained high alkalinity for longer periods of time than Ca(OH)_2_ powder (Fig. 2d). This result suggests that granulation with zeolite provides advantages, by preventing carbonation caused during the preparation process. By optimizing the preparation process, it will be possible to SLBGs with higher Ca(OH)_2_ content and longer-lasting high alkalinity.

### Water is required for the disinfection activity of Ca(OH)_2_

**Figure 3:**
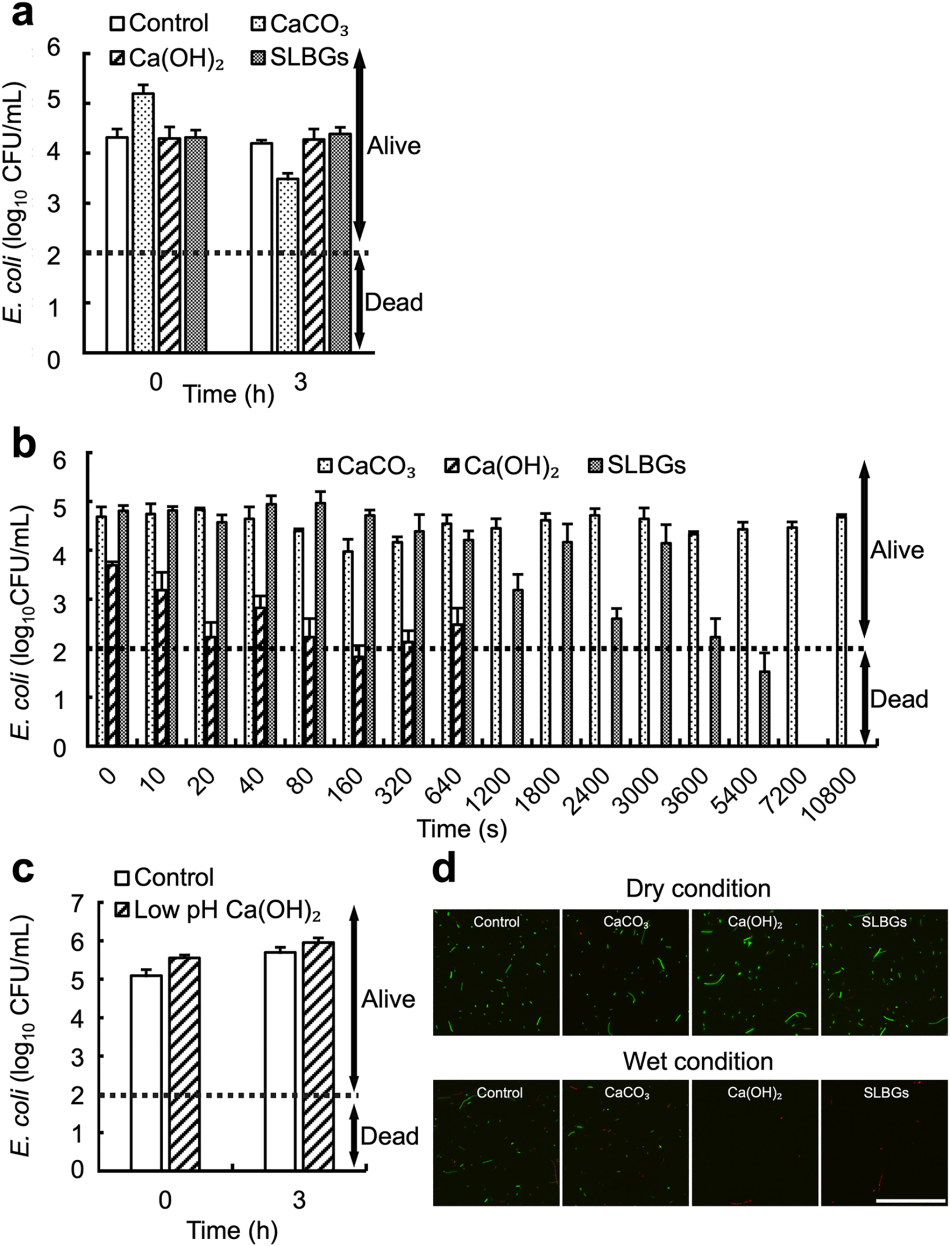
The effect of water on disinfection. (**a**) *E. coli* abundance (log_10_ colony-forming units, CFU) after contact without (control) or with disinfectant (CaCO_3_, CA(OH)_2_, or SLBGs) with no water added (dry condition) for 3 h. The dashed line indicates the detection limit estimated from the dilution ratio. (**b**) *E. coli* abundance after contact with disinfectant (CaCO_3_, Ca(OH)_2_, or SLBGs) with water added (wet condition) for up to 3 h. (**c**) *E. coli* abundance after contact without (control) with low pH Ca(OH)_2_ (in this Ca(OH)_2_, pH was reduced to about 9.28 in the door test for 63 days in Fig. 2d) with water added (wet condition) for up to 3 h. (**d**) Live/dead assay with disinfectant (control, CaCO_3_, Ca(OH)_2_, or SLBGs) in dry and wet conditions after 3 h. Images were captured using a fluorescence microscope equipped with a 20 × objective. Bar, 100 μm. Data in **a**, **b**, and **c** represent the mean and SD (error bars) from three separate experiments. Alive and Dead in **a**, **b**, and **c** indicate the range of CFU in which the number of colonies was observed and the range of CFU below the detection limit (2 log_10_ CFU/mL) in which no colonies were observed, respectively.

To directly evaluate the disinfectant activity associated with the pH changes induced by the Ca(OH)_2_ treatments, we investigated in vitro bactericidal effects. In this study, *E. coli*, which is highly resistant to Ca(OH)_2_^6–8^, was used as a model pathogen to evaluate the disinfectant activity of the treatment. In previous studies, *E. coli* and *Salmonella* underwent a 6 log_10_ and 4 log_10_ decrease, respectively, after 10 min of exposure to a Ca(OH)_2_ solution in an in vitro experiment^7^, suggesting that Ca(OH)_2_ is an effective disinfectant. However, in that report^7^, it was unclear whether Ca(OH)_2_ exerted its disinfectant effect under stand-by disinfection conditions (Supplementary Fig. 1, Fig. 2) because the evaluation was performed using Ca(OH)_2_ solution^7^. Moreover, a separate study reported that after Ca(OH)_2_ powder was added to livestock feces, that the powder became inactive against *E. coli* and *Salmonella* within 6 h^16^. However, water was also abundant in that system, while the inactivation was due to some other factors such as CO_2_ emission by microbes in the feces. Therefore, we developed a new method (Supplementary Fig. 3) to evaluate the disinfection effects of dry Ca(OH)_2_ powder, SLBGs, and CaCO_3_ powder on *E. coli* under stand-by disinfection conditions. Details of the new method and its verification are explained in the Supplementary methods and Supplementary Figs. 4–8, respectively. At the initiation of contact between the disinfectants and *E. coli*, bacterial abundance was 4–5 log_10_ colony-forming units (CFU)/mL in all samples (Fig. 3a, 0 h). After 3 h of contact, abundance was similar in all samples, including the control (i.e., without any disinfectants) sample (Fig. 3a, 3 h). These results (Fig. 3a) clearly demonstrate that dry Ca(OH)_2_ could not inactivate *E. coli*, indicating that Ca(OH)_2_ disinfectant activity requires water.

**Figure 4:**
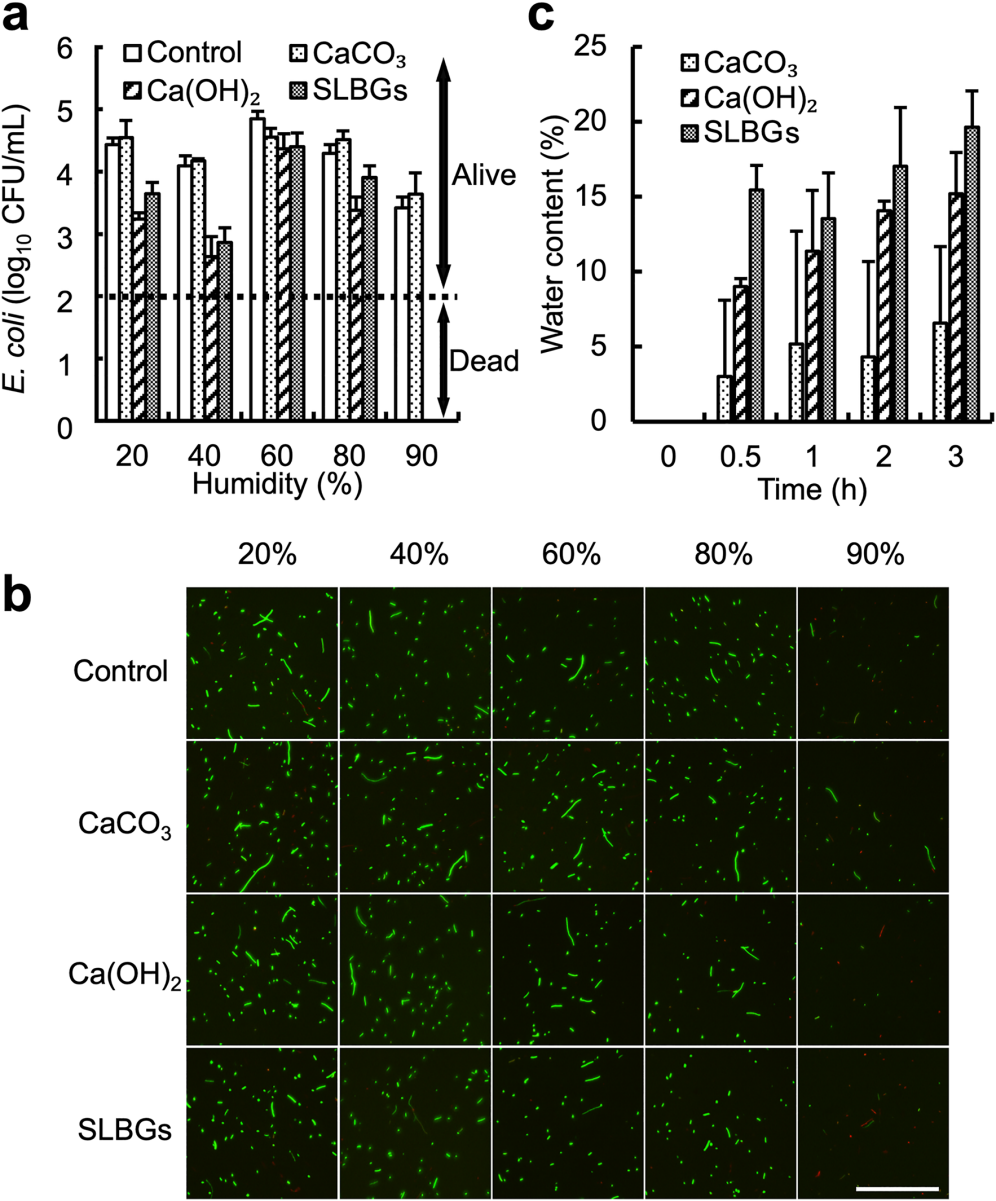
The effect of humidity on disinfection. (**a**) *E. coli* abundance (log_10_ CFU) after contact with disinfectant (control, CaCO_3_, Ca(OH)_2_, or SLBGs) under various humidity conditions (20%, 40%, 60%, 80%, or 90%) for 3 h. The detection limit was 2 log_10_ CFU/mL (dashed line). (**b**) Live/dead assay with the disinfectant (control, CaCO_3_, Ca(OH)_2_, or SLBGs) under various humidity conditions (20%, 40%, 60%, 80%, or 90%) after 3 h. Images were captured using a fluorescence microscope equipped with a 20 × objective. Bar, 100 μm. (**c**) Water content of disinfectant (CaCO_3_, Ca(OH)_2_, or SLBGs) over time at 35 °C and 90% humidity. Data in **a** and **c** represent the mean and SD (error bars) from three separate experiments.

Next, we evaluated the disinfection effect when water was added under the same conditions (Fig. 3b). The abundance of *E. coli* was approximately 4–5 log_10_ CFU/mL before incubation (Fig. 3b, 0 s). After exposure to wet CaCO_3_ powder for 3 h in the presence of water (5 μL of water per 1.5 mg of CaCO_3_), the abundance of *E. coli* was 4.7 log_10_ CFU/mL (Fig. 3b, 10,800 s), suggesting that CaCO_3_ did not exert a disinfection effect, even in the presence of water (Fig. 3b, dotted bars). This result revealed that a weakly alkaline (~ pH 9) CaCO_3_ solution had no disinfecting effect on *E. coli*. We also confirmed that Ca(OH)_2_, whose pH dropped to 9.28 in the door test after 63 days, had no disinfecting effect (Fig. 3c, diagonal bars). Upon exposure to Ca(OH)_2_ powder in the presence of water, the abundance of *E. coli* was reduced to 2.5 log_10_ CFU/mL in 640 s and decreased below the detection limit after 1,200 s of contact (Fig. 3b, diagonal bars). After the addition of SLBGs to *E. coli* in the presence of water, the bacterial abundance decreased to 2.2 log_10_ CFU/mL in 3600 s, to 1.5 log_10_ CFU/mL in 5400 s, and below the detection limit after 7,200 s (Fig. 3b, gray bars). These results (Fig. 3a,b) confirm that disinfection by Ca(OH)_2_ requires the presence of water. Furthermore, monitoring the potential environmental degradation of Ca(OH)_2_ to CaCO_3_ is also important to ensure adequate stand-by disinfection, because CaCO_3_ (Fig. 3b) and Ca(OH)_2_ with reduced pH (Fig. 3c) did not show disinfection activity under this degraded condition.

Next, we performed a live/dead assay to confirm whether disinfection occurred concomitantly with the changes in pH under the same conditions. In this experiment, live and dead *E. coli* were stained green and red using DMAO and EthD-III solutions in Bacteria Live/Dead Staining Kit, respectively. DMAO is a green fluorescent dye that stains both live and non-membrane-damaged bacteria, and EthD-III is a red fluorescent dye that stains only dead bacteria with membrane damage^17^. *E. coli* were stained green in the presence of each disinfectant in the dry condition (Fig. 3d, dry condition), suggesting that *E. coli* survived dry conditions (Fig. 3a). In the wet condition, *E. coli* also stained green in the control and CaCO_3_ samples (Fig. 3d, wet condition). In contrast, *E. coli* treated with Ca(OH)_2_ powder or SLBGs were stained red (Fig. 3d, wet condition), suggesting that these disinfectants were effective in wet conditions. We then confirmed the presence of *E. coli* in dry conditions by microscopic analysis of all samples (Supplementary Fig. 9), and also confirmed that the abundance of *E. coli* decreased after exposure to Ca(OH)_2_ powder or SLBGs in wet conditions (Supplementary Fig. 9). One possible explanation for these results is that the cell wall/membrane was disrupted by high alkalinity in wet conditions in the presence of Ca(OH)_2_ powder or SLBGs. Ca(OH)_2_ solution can inactivate bacteria by effectively destroying the cell wall/membranes^18^, supporting this result.

### High levels of humidity mediate Ca(OH)_2_ disinfectant activity

Stand-by disinfection is performed under variable environmental conditions. Many water molecules are adsorbed to the surface of Ca(OH)_2_ particles under high humidity, which may contribute to the disinfection effect. Two studies reported that Ca(OH)_2_ powder exhibited disinfection effects under moist conditions, although the exact moisture content was not reported^19,20^. To investigate the effect of humidity on disinfection activity under stand-by disinfection conditions, we evaluated the viability of *E. coli* at 20%, 40%, 60%, 80%, and 90% humidity using thermo-hygrostat chamber (Fig. 4a). Soil surface temperature is approximately 5 °C higher than air temperature^21^, so we performed this evaluation at 35 °C, to represent the summer ground temperature in Japan. When *E. coli* was exposed to the disinfectants for 3 h, colonies were detected in all samples at 20%, 40%, 60%, and 80% humidity (Fig. 4a). However, at 90% humidity, colonies were not detected in the Ca(OH)_2_ powder (Fig. 4a, diagonal bars) and SLBG (Fig. 4a, gray bars) samples but were detected in the control (Fig. 4a, white bars) and CaCO_3_ (Fig. 4a, dotted bars) samples, suggesting that these two disinfectants were effective under high humidity (~ 90%), without water being present in a liquid phase.

Figure 4b shows the results of a confirmatory live/dead assay. *E. coli* stained green under all conditions in the control, CaCO_3_ powder, Ca(OH)_2_ powder, and SLBG samples at 20%, 40%, 60% and 80% humidity. However, at 90% humidity, *E. coli* stained green in the control and CaCO_3_ powder samples but red in the Ca(OH)_2_ powder and SLBG samples, again suggesting that the latter two disinfectants are effective at 90% humidity. Microscopy observation of *E. coli* (Supplementary Fig. 10) suggest that *E. coli* was inactivated without any change in cell morphology. This result implies that contact with the solid Ca(OH)_2_ surface holding water partially destroyed the cell wall/membrane of *E. coli*, resulting in cytoplasmic leakage and cell death.

To determine how much water adsorption is required for the observed effect, we also measured the amount of water adsorbed by the samples at 90% humidity (Fig. 4c). After 0.5 h of incubation, adsorbed water accounted for 3.0% of the CaCO_3_ mass (Fig. 4c, dotted bars), 9.0% of the Ca(OH)_2_ mass (Fig. 4c, diagonal bars) and 15% of the SLBG mass (Fig. 4c, gray bars). After 3 h of incubation, when the disinfection effect was confirmed (Fig. 4c), 6.6%, 15%, and 20% water had adsorbed to the CaCO_3_, Ca(OH)_2_, and SLBGs, respectively. Zeolite, one of the raw materials of SLBGs, is an aluminosilicate crystal composed of SiO_4_ and AlO_4_ in a tertiary structure that is also used for the removal of ammonia and CO_2_^22–24^. The adsorption of water to SLBGs may have been higher than to Ca(OH)_2_ since zeolite adsorbs water due to its hygroscopicity. Overall, these results (Fig. 4) reveal that 15–20%wt of water content in Ca(OH)_2_ or SLBG disinfectant is necessary for disinfection. These results indicate potential best practices regarding the use of Ca(OH)_2_ in the field.

### On-site detection of the disinfection effect by a pH indicator

**Figure 5:**
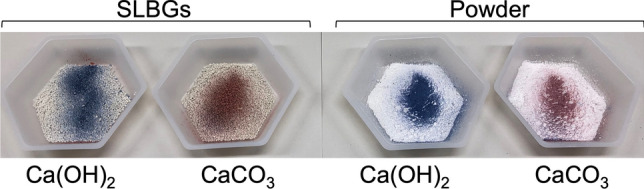
Detection of carbonation of Ca(OH)_2_ using a pH indicator. SLBGs containing Ca(OH)_2_ or CaCO_3_ (left) or powder consisting of Ca(OH)_2_ or CaCO_3_ (left) (right) were sprayed with the pH indicator we developed (Uwai et al., manuscript in preparation). Granules and powder containing Ca(OH)_2_ instantly turned blue from red due to their strong alkalinity, but granules and powder containing CaCO_3_ remained red (Supplementary Movie 1).

Since the persistence of pH as a basis for the disinfection effect (Fig. 3c) varies depending on the situation of stand-by disinfection and on the type of Ca(OH)_2_ disinfectant (Fig. 2), it is important to periodically evaluate the disinfection activity using, in this case, the persistence of, or changes to, pH as a proxy. However, periodic pH measurement using a pH meter is time consuming and expensive, so we developed a simple evaluation method using a pH indicator. The detailed preparation method of the pH indicator will be reported elsewhere (Uwai et al., manuscript in preparation), but the results obtained using the pH indicator are shown in Fig. 5 and Supplementary Movie 1. Spray application of the pH indicator stained the SLBGs prepared from Ca(OH)_2_ and Ca(OH)_2_ powder blue, but the SLBGs prepared from CaCO_3_ and CaCO_3_ powder stained red (Fig. 5). With this spray, farmers will be able to quickly determine the degradation of Ca(OH)_2_ and make necessary adjustments. In addition, if farmers have overspread Ca(OH)_2_, this adjustment may save using it.

## Conclusion

In this study, we found that the pH of Ca(OH)_2_ decreases within about two weeks to one month under stand-by disinfection conditions and loses its disinfecting activity while 15–20% (w/w) water is required for the disinfecting effect. We further confirmed that the pH persistence of SLBGs was more than twice as long as Ca(OH)_2_ powder. These results will help to establish an appropriate application of Ca(OH)_2_ to protect farms from infectious diseases.

## Methods

### Materials

Ca(OH)_2_ powder was purchased from Hokkaido Lime Co., Ltd. (Tomakomai, Japan), CaCO_3_ powder was purchased from Kanto Chemical Co., Inc. (Tokyo, Japan), and zeolite was purchased from Hokkaido Zeolite Co., Inc. (Otaru, Japan). Ca(OH)_2_ powder and SLBGs were subdivided into screw cap tubes or plastic bags and stored in a sealed state.

### Preparation of slaked lime-based granules (SLBGs)

SLBGs were prepared using a stirring granulator (WB-5, Pacific Machinery & Engineering, Narashino, Japan). First, 750 g of Ca(OH)_2_ powder and 750 g of zeolite were premixed for 60 s in the granulator. After 500 mL of deionized water was added to the mixed powder, granulation was carried out for 300 s, using spindle and chopper rotation speeds of 167 and 3000 rpm, respectively. The granules were dried in an oven at 60 °C overnight. In this study, granules of 1.0 to 2.0 mm were used for the biological tests. The effects of granulation conditions on the physical properties of the granules (namely, granule size distribution and hardness) are described in detail elsewhere^13^.

### Evaluation of the persistence of pH changes induced by disinfectants

Ca(OH)_2_ powder was used for stand-by disinfection at four outdoor locations (soil, grass, road, and in front of a barn, Supplementary Fig. 1). For stand-by disinfection, 0.5–1 kg/m^2^ Ca(OH)_2_ powder was spread, such that 1 kg of Ca(OH)_2_ powder for 1 m^2^ was spread at the four outdoor locations (Fig. 2a). To measure pH, 1 g of disinfectant was collected in a 15 mL plastic tube, 2.5 mL of distilled water was added, and the mixture was stirred. The suspension was left for 1 h, and the pH of the supernatant was measured by a pH meter (Docu-pH meter, Sartorius, New York, USA).

For the indoor experiment, a paper box was placed on artificial plastic grass (440–0011, Mizushima, Osaka, Japan), and 62.5 g of disinfectant was spread on the paper box (Fig. 2c). Water was sprayed daily (200 cm^3^/day) to match the annual precipitation in Hokkaido, Japan^25^. This amount was determined as follows. Since average rainfall in Hokkaido from 1986 to 2015 was 114.8 cm/year (0.315 cm/day) and the size of the paper box was 25 × 25 cm, the daily watering rate was set to 25 × 25 × 0.32 cm = 200 cm^3^. Water (200 cm^3^) was applied once a day using a watering can. pH was measured in the same way as in the outdoor experiment.

### Evaluation of the disinfection effect under stand-by disinfection conditions

Our method to evaluate the effectiveness of disinfection (Supplementary Fig. 3) was established according to the conditions described in Supplementary Figs. 6–10. One milliliter of bacterial suspension was dispensed into a 1.5 mL plastic tube and centrifuged (9,100 g, 2 min, 4 °C). Then, the supernatant was removed. The pellet was resuspended in 1 mL of phosphate-buffered saline (PBS) and centrifuged (2,300 g, 2 min, 4 °C), and the supernatant was removed again. The obtained pellets were dried in an incubator (37 °C, 3 h). To increase the contact area with the disinfectant, the dried cells were homogenized for 5 s with a Powermasher II (Nippi, Tokyo, Japan). Disinfectant (1.5 mg) was added to the homogenized dried cells, vortexed for 3 s and statically incubated for various periods. After incubation, the homogenized samples were instantly neutralized by adding 1 mL of 0.1 M phosphate buffer (pH 6.8). The neutralized solution was then diluted tenfold and 100-fold. Then, 0.1 mL of the diluted samples were applied to plastic Petri dish (90 × 20 mm), and the number of colonies was counted after overnight incubation at 37 °C. Finally, *E. coli* log_10_ number of CFUs was determined.

### *Escherichia coli* strains

Since *E. coli* is the most commonly used gram-negative bacterial model for pathogenic bacteria^26^, *E. coli* XL-1 blue was used as the model bacterium in this study. *E. coli* XL-1 blue was purchased from GMbiolab Co., Ltd. (Taichung, Taiwan). First, 100 µL of *E. coli* XL-1 blue that had been stored at − 80 °C was thawed and precultured (37 °C, 80 rpm, overnight) in LB medium (10 g/L polypeptone, 5 g/L yeast extract, 5.0 g/L NaCl, pH 7.0). Next, a primary culture (LB medium, 37 °C, 80 rpm, 15 h) was inoculated with 100 µL of the preculture in LB medium. The primary culture after the logarithmic growth phase, in which the number of viable bacteria was maximized, was used for the experiment.

### Evaluation of disinfection in the presence of water

For the dry condition, 1.5 mg of disinfectant was added to the homogenized dry cells in a 1.5 mL tube, which was then closed. For the wet condition, 1.5 mg of disinfectant and 5 µL of water were added to the homogenized dry cells, and the tube was closed and statically incubated for various periods. For the controlled-humidity conditions, 1.5 mg of disinfectant was added to the homogenized dry cells, and the tube was placed in a thermo-hygrostat chamber (SH-222, Espec, Osaka, Japan) for 3 h. In this incubator, the 1.5 mL tube was opened. After incubation, the sample was neutralized instantly by adding 1 mL of 0.1 M phosphate buffer. The resulting neutralized solution was diluted tenfold and 100-fold with 0.1 M phosphate buffer, 0.1 mL of the diluted sample was applied to a plate containing LB agar medium, and the number of colonies was counted after incubation. The *E. coli* log_10_ number of CFUs was then determined.

### Fluorescence microscopy

The disinfection effect was confirmed using a Bacteria Live/Dead Staining Kit (Takara Bio Inc., Kusatsu, Japan). After the samples were statically incubated with disinfectant, the disinfectant was neutralized with 1 mL of 0.1 M phosphate buffer. The desired sample dilution was obtained by adding 60 µL of 0.1 M phosphate buffer to 40 µL of the neutralized suspension. Next, 1 µL of staining solution (DMAO:EthD-III:0.85% NaCl = 1:2:8) was added to the diluted solution, which was then incubated in the dark (room temperature, 15 min). Images were captured using a conventional fluorescence microscope (ECLIPSE Ni; Nikon, Tokyo, Japan) equipped with a CMOS camera (DS-Qi2; Nikon) and an objective lens (Plan Fluor ELWD 20 × /0.45, Nikon) together with NIS-Elements ver. 4.5 software (Nikon). All procedures were performed at room temperature (22 ± 2 °C). Immunofluorescence images were analyzed using NIS-Elements software (Nikon) and Image J ver. 1.52a (NIH, Bethesda, USA).

### Water adsorption by the disinfectant

To determine the water content adsorbed by the disinfectant at 35 °C and 90% humidity, 1.5 mg of disinfectant was placed in a 1.5 mL tube and incubated in a thermo-hygrostat chamber (SH-222, Espec). Then, the weight of the tube was measured. The water content was determined as the ratio of the weight of water adsorbed on the disinfectant to the weight of the disinfectant that adsorbed the water.

### Preparation of the pH indicator

The preparation of the pH indicator for the on-site assessment of Ca(OH)_2_ pH indicator is described elsewhere (Uwai et al., manuscript in preparation).

### Statistics

Evaluations of the persistence of pH changes, disinfection effects, and water absorption were performed in three independent experiments (triplicate of each experiment). The data were analyzed using Excel software (Microsoft, Redmond, USA). In this paper, we defined CFU below the detection limit (2 log_10_ CFU/mL) as a significant disinfectant effect.

## Supplementary Information

Supplementary Information 1.

Supplementary Information 2.
