# Supplementary material for: Farm use of calcium hydroxide as an effective barrier against pathogens
Source: Sci Rep. 2021 Apr 12;11:7941. doi: 10.1038/s41598-021-86796-w (PMC8041792; doi:10.1038/s41598-021-86796-w)
Supplement: Supplementary file 1 — Supplementary Information 1. [file 41598_2021_86796_MOESM1_ESM.pdf]

Supplementary Information

Farm use of calcium hydroxide as an effective barrier against pathogens

Shinji Matsuzaki, Kento Azuma, Xuguang Lin, Masahiro Kuragano, Koji Uwai, Shinya Yamanaka*, and Kiyotaka Tokuraku*

Department of Applied Science, Muroran Institute of Technology, Muroran, Hokkaido, Japan

*Corresponding authors:

Dr. Shinya Yamanaka, e-mail: syama@mmm.muroran-it.ac.jp

Dr. Kiyotaka Tokuraku, e-mail: tokuraku@mmm.muroran-it.ac.jp

This file includes:

(1) Supplementary method: Development of a method for evaluation of disinfection under stand-by disinfection conditions

(2) Supplementary Figs. 1–10

(1) Supplementary method: Development of a method for evaluation of disinfection under stand-by disinfection conditions

Our method of evaluating disinfection under stand-by disinfection conditions consisted of the following four steps (Supplementary Fig. 4): (1) preparation of the bacteria and disinfectants, (2) contact between the bacteria and disinfectants, (3) neutralization of disinfectants, and (4) evaluation of disinfection by the dilution plate method and live/dead assay.

(1) Bacterium/disinfectant preparation

As mentioned in the main text, one of the benefits of stand-by disinfection with calcium hydroxide ($Ca(OH)_2$) is that it can be spread directly on the ground without the use of a cistern. Thus, solid $Ca(OH)_2$, and not $Ca(OH)_2$ in solution, enters into contact with the pathogen. Thus, to develop an assay to accurately replicate these conditions, we first examined the culture and drying conditions of *E. coli* (XL-1 blue), which was used as the model pathogen. Supplementary Fig. 5 shows the growth curve of *E. coli* and $\log_{10}$ CFU numbers determined by the dilution plate method. Absorbance at 600 nm, which correlates with the number of bacterial cells, began to increase after 4 h of culture and reached a plateau between 15 and 21 h (Supplementary Fig. 5a). Comparison of incubation for 15, 18, and 21 h revealed that the *E. coli* CFU reached an approximate maximum after 15 h of incubation (Supplementary Fig. 5b). Therefore, the culture time used in our assay was 15 h.

To prepare live *E. coli* in the solid state, we next examined the relationship between drying and viability (Supplementary Fig. 6). Since the dryness of *E. coli* reached equilibrium after drying for more than 2 h, the drying time of *E. coli* was set to 3 h.

(2) Contact between bacteria and disinfectant

To ensure contact between solid $Ca(OH)_2$ and dried *E. coli*, control of the contact area is important. Here, the dried *E. coli* was refined using a homogenizer for approximately 10 s (Supplementary Fig. 7a top) so that the particle diameter of the dried *E. coli* (Supplementary Fig. 7a bottom) was almost the same as that of the calcium carbonate ($CaCO_3$) powder (~10 μm). We did not identify any difference in viability caused by

homogenization (Supplementary Fig. 7b). The SLBGs were also refined with a homogenizer to yield the same size as the $Ca(OH)_2$ powder. The refined *E. coli* pellet and 1.5 mg disinfectant powder were then mixed together by vortexing, before being placed in contact with each other under various conditions and for different amounts of time.

(3) Neutralization of disinfectants

In general, the dilution plate method is used to measure the number of viable bacteria. Since the dilution plate method involves dilution in an aqueous solution, it was necessary to instantly neutralize the strong alkalinity of $Ca(OH)_2$ in order to accurately count viable *E. coli*. Therefore, we examined neutralization using PBS and 0.1 M phosphate buffer (Supplementary Fig. 8). Phosphate buffer (0.1 M) was used because PBS did not neutralize $Ca(OH)_2$ under the conditions of this experiment.

(4) Evaluation of the disinfection effect by the dilution plate method

The neutralized solution was evaluated by the dilution plate method. The experiment without incubation in the wet condition using $Ca(OH)_2$ (Fig. 3b, 0 h) showed the same number of colonies as the control sample (Fig. 3a, 0 h), suggesting that neutralization and evaluation by the dilution plate method were performed accurately.

(2) Supplementary Figs. 1–10

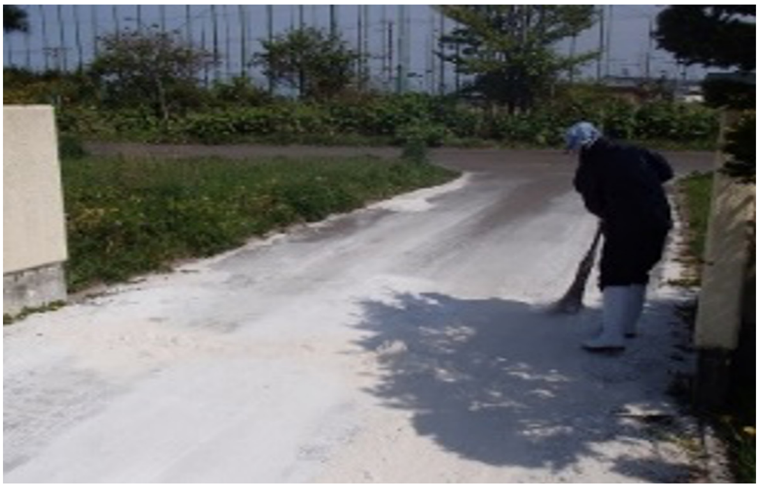

Supplementary Fig. 1. Stand-by disinfection. The white powder spread by a broom is $Ca(OH)_2$. $Ca(OH)_2$ powder (0.5–1.0 kg/m^2) was widely spread at the entrance to a farm and farm-related facilities. The picture shows the entrance to a livestock hygiene service center in Hokkaido, Japan.

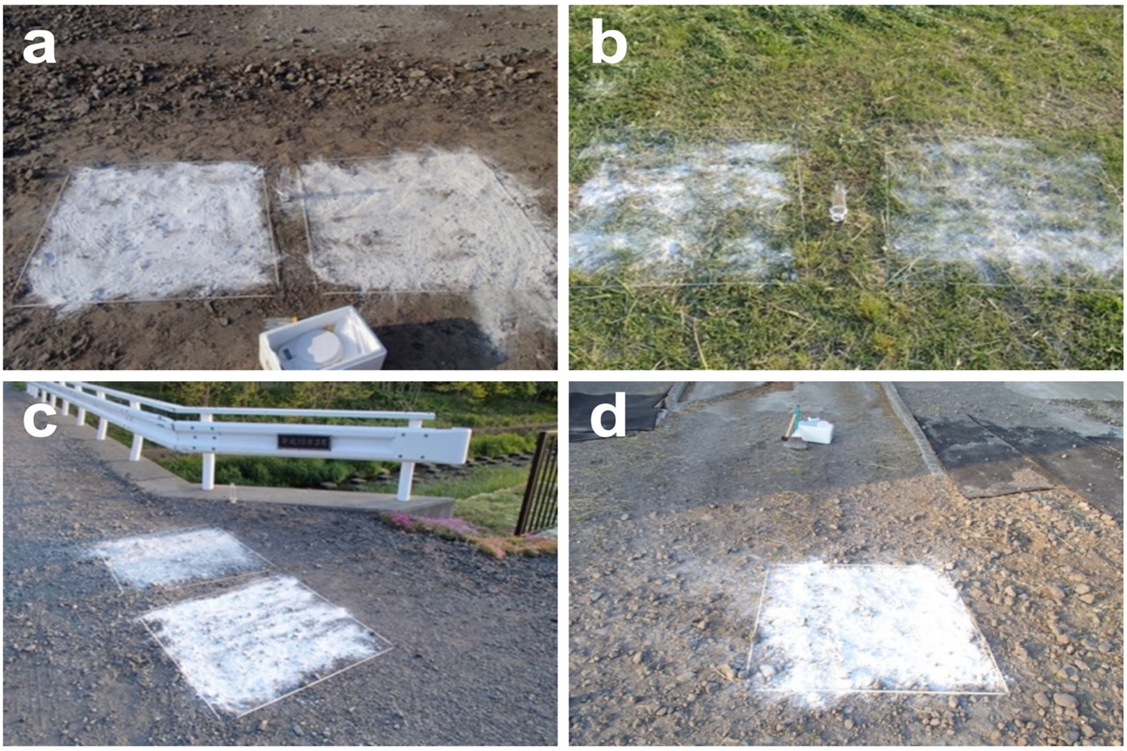

Supplementary Fig. 2. Evaluation of pH persistence under stand-by disinfection conditions at outdoor locations in Hokkaido, Japan. One kg of $Ca(OH)_2$ powder was spread over 1 m^2 at the four outdoor locations, on soil (**a**), grass (**b**), a road (**c**), and in front of a barn (**d**). **c** and **d** are the pathways of vehicles and people, but **a** and **b** are not. There are two scattered areas in the photographs, but one area is another experiment unrelated to this paper. Samples were taken independently from different three points in each area and the pH was measured.

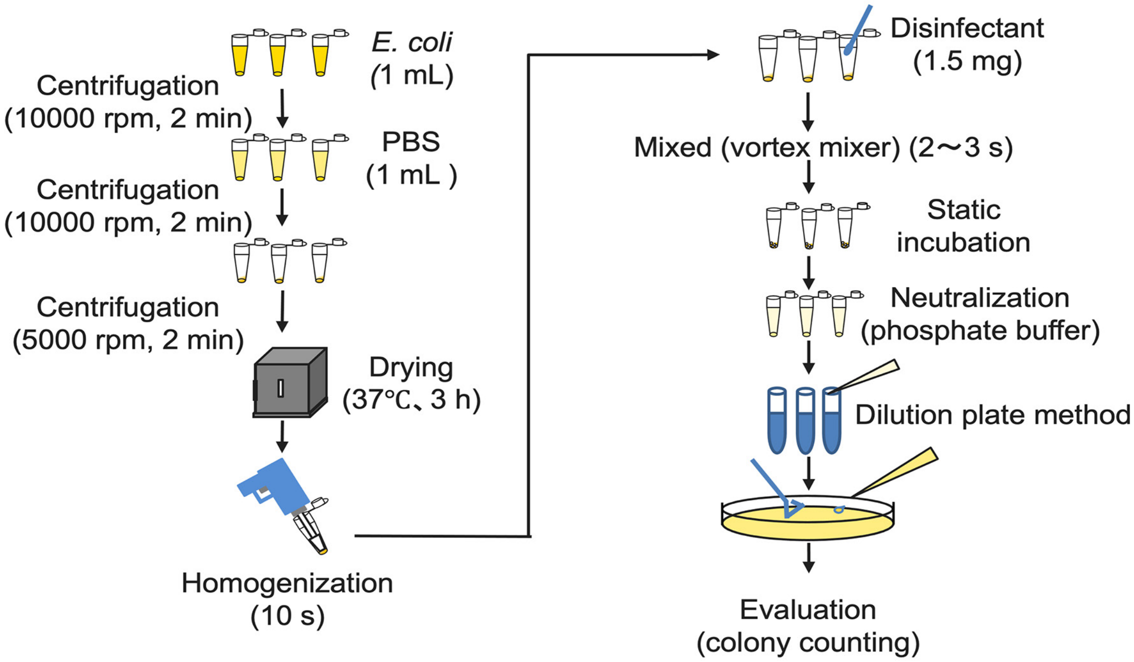

Supplementary Fig. 3. Scheme of the disinfection evaluation method under conditions equivalent to stand-by disinfection conditions. One mL of the cultured *E. coli* XL-1 blue (LB medium, 37 °C, 80 rpm, 15 h) was transferred to a 1.5 mL tube, and centrifuged at 10,000 rpm (9,100 *g*) for 2 min. The medium was removed, and 1 mL of PBS was added and the pellet was resuspended. The suspension was centrifuged at 10,000 rpm (9,100 *g*) for 2 min, and supernatant was removed by decantation. The tube was centrifuged at 5,000 rpm (2,300 *g*) for 2 min, and remaining PBS was removed with a micropipette. The *E. coli* was dried at 37 °C for 3 h in an incubator and homogenized for 10 s. 1.5 mg of disinfectant was added to the homogenized dry cells in a 1.5 mL tube and mixed by a vortex mixer for a few seconds. The tube was incubated statically in a thermo-hygrostat. After the reaction, the sample was neutralized instantly by adding 1 mL of a 0.1 M phosphate buffer. The resulting neutralized solution was diluted 10-fold and 100-fold with 0.1 M phosphate buffer, 0.1 mL of the diluted sample was applied to a plate, and the number of colonies was counted after incubation. This method was established according to the conditions shown in Supplementary Figs. 6-10.

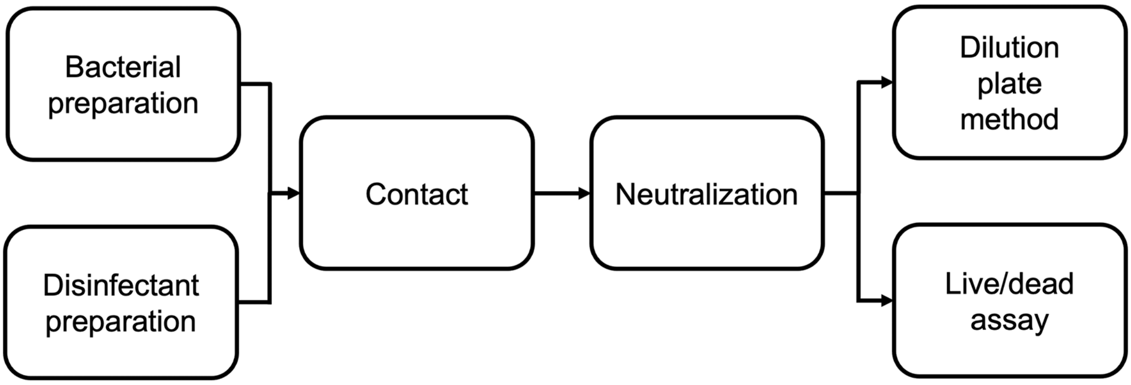

Supplementary Fig. 4. Scheme describing the method to evaluate the disinfection effect under stand-by disinfection conditions.

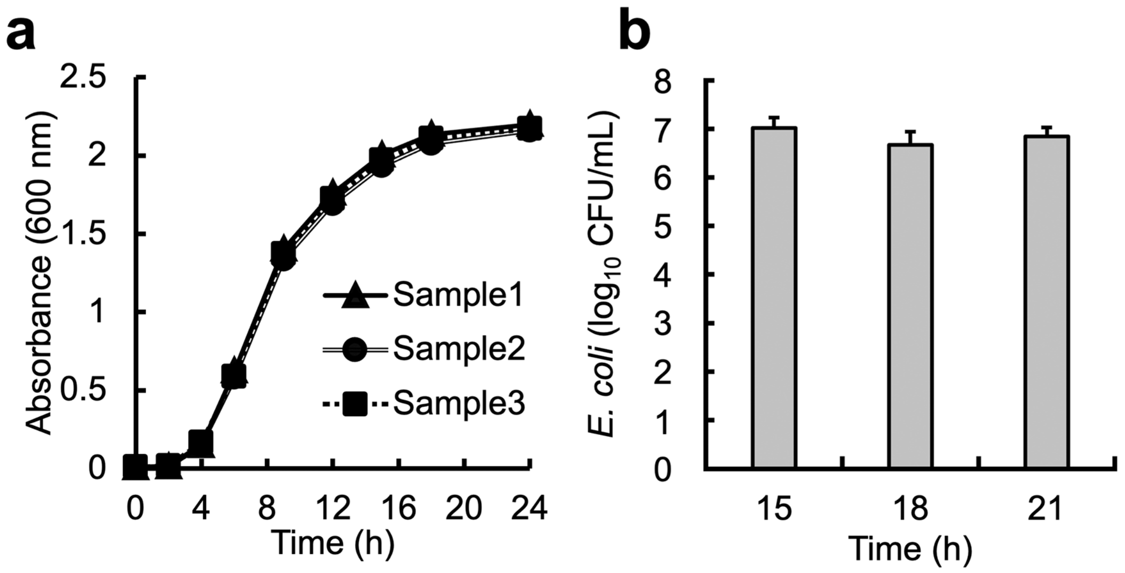

Supplementary Fig. 5. Growth curve of *E. coli* and *E. coli* log$_{10}$ number of CFUs on solid medium. (a) *E. coli* XL-1 blue stock (100 µL) that had been stored at -80 °C was thawed and precultured (37 °C, 80 rpm, overnight) using LB medium. Next, the primary culture (LB medium, 37 °C, 80 rpm, 15 h) was inoculated using 100 µL of the preculture. One milliliter of *E. coli* cultured in LB medium was collected at each time point, and the absorbance at 600 nm was measured with a spectrophotometer (Ultrospec 1100 pro, GE Healthcare, Chicago, IL, USA). Each sample represents the mean of three separate experiments. **(b)** After culturing *E. coli* in LB medium for the specified period, the medium was removed by centrifugation (10,000 rpm, 2 min). Next, the pellet was suspended in PBS and serially diluted. Then, 0.1 mL of the diluted solution was inoculated on an LB plate, and after incubation overnight, the number of colonies was counted. Data represent the mean and SD (error bars) from three separate experiments.

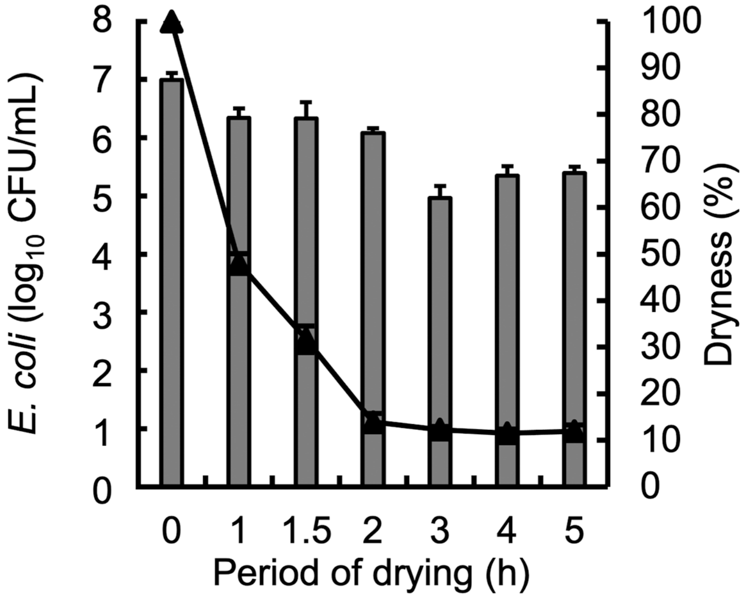

Supplementary Fig. 6. *E. coli* $\log_{10}$ CFU numbers and dryness when *E. coli* was dried for different periods of time. The horizontal axis represents the drying period of *E. coli*. The bar graph represents the *E. coli* $\log_{10}$ number of CFUs. The line graph represents the dryness of the *E. coli* pellet. Dryness is the percentage of weight after drying relative to the weight before drying. The pelleted *E. coli* was dried at 37 °C in an incubator to obtain dried cells. Data represent the mean and SD (error bars) from three separate experiments.

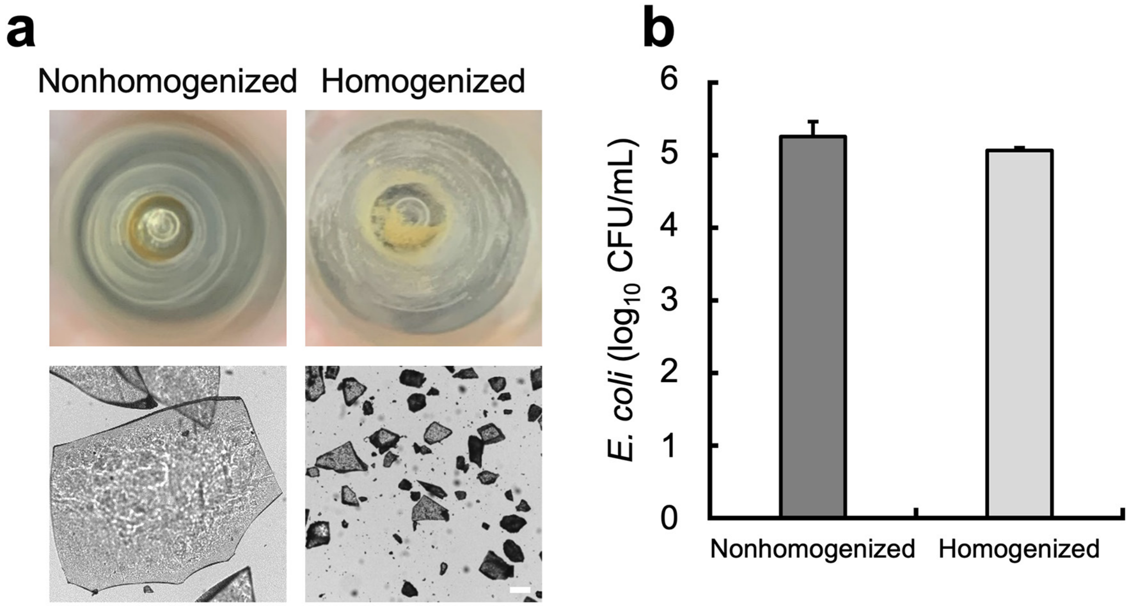

Supplementary Fig. 7. Effect of dry cell homogenization on the *E. coli* $\log_{10}$ number of CFUs. Samples were prepared by homogenizing the dried cells with a BioMasher or without homogenization for 10 s, the cells were suspended in 0.1 M phosphate buffer, and the *E. coli* $\log_{10}$ number of CFUs were evaluated. **(a)** The upper row includes photographs of dried cells before (left) and after (right) homogenization. The bottom row is a bright-field microscope image (4x objective) of the dried cells before (left) and after (right) homogenization. Scale bar represents 100 µm. **(b)** *E. coli* $\log_{10}$ CFU numbers of nonhomogenized or homogenized cells. Data represent the mean and SD (error bars) from three separate experiments.

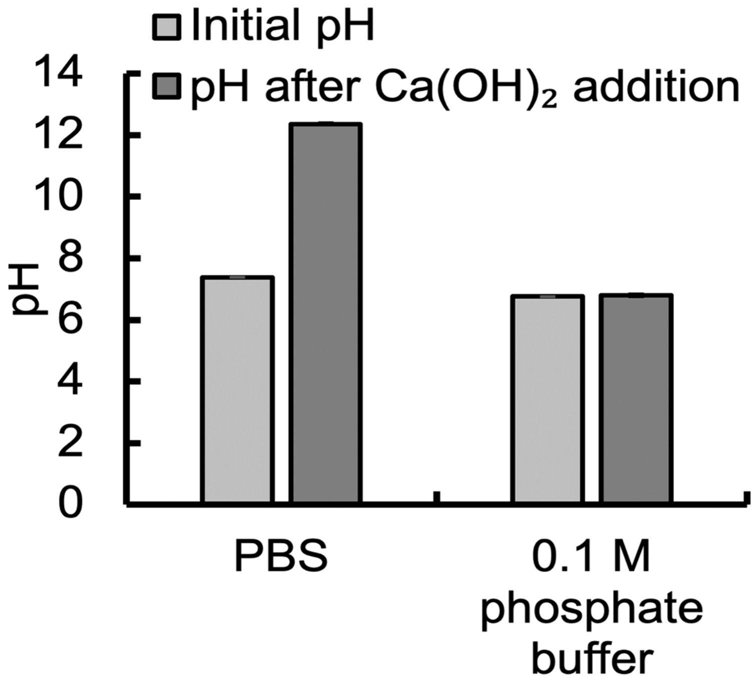

Supplementary Fig. 8. Buffering capacity of PBS and 0.1 M phosphate buffer against $Ca(OH)_2$ powder. The initial pH of PBS and 0.1 M phosphate buffer was measured. Next, the pH was measured when $Ca(OH)_2$ powder was added to the same concentration as that used in the experiment. Data represent the mean and SD (error bars) from three separate experiments. Error bars are not visible due to small variations.

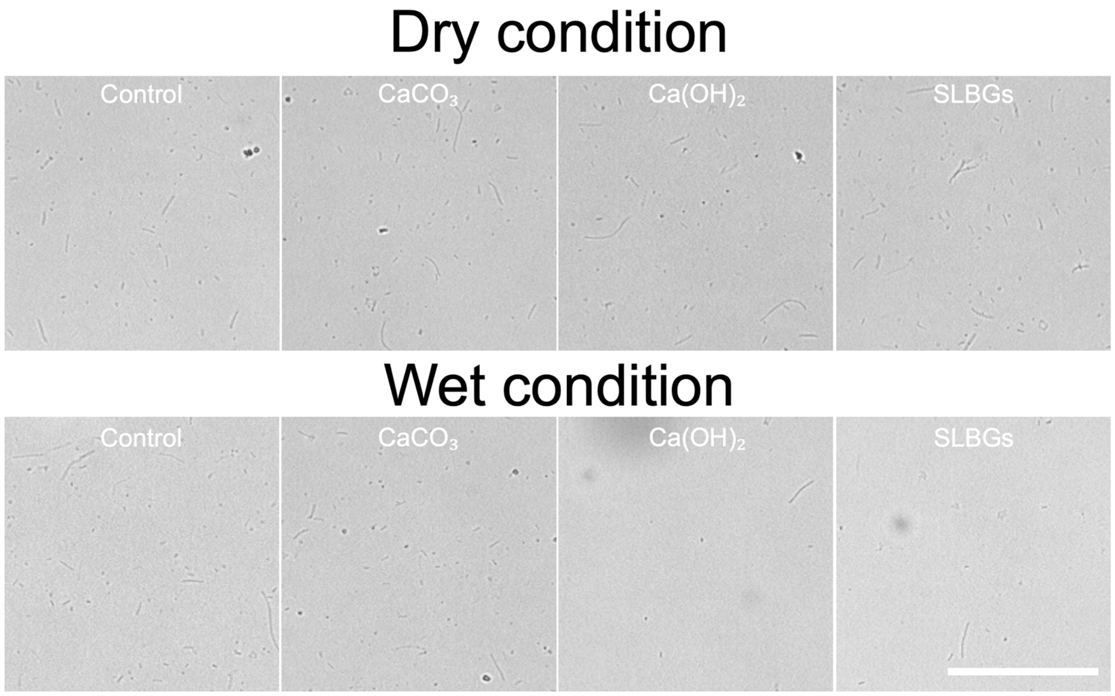

Supplementary Fig. 9. Bright-field images of *E. coli* treated with each disinfectant (control, $CaCO_3$, $Ca(OH)_2$, or SLBGs) under dry and wet conditions for 3 h (the same condition as in Fig. 3d). Images were captured using a fluorescence microscope equipped with a 20x objective. Bar, 100 µm.

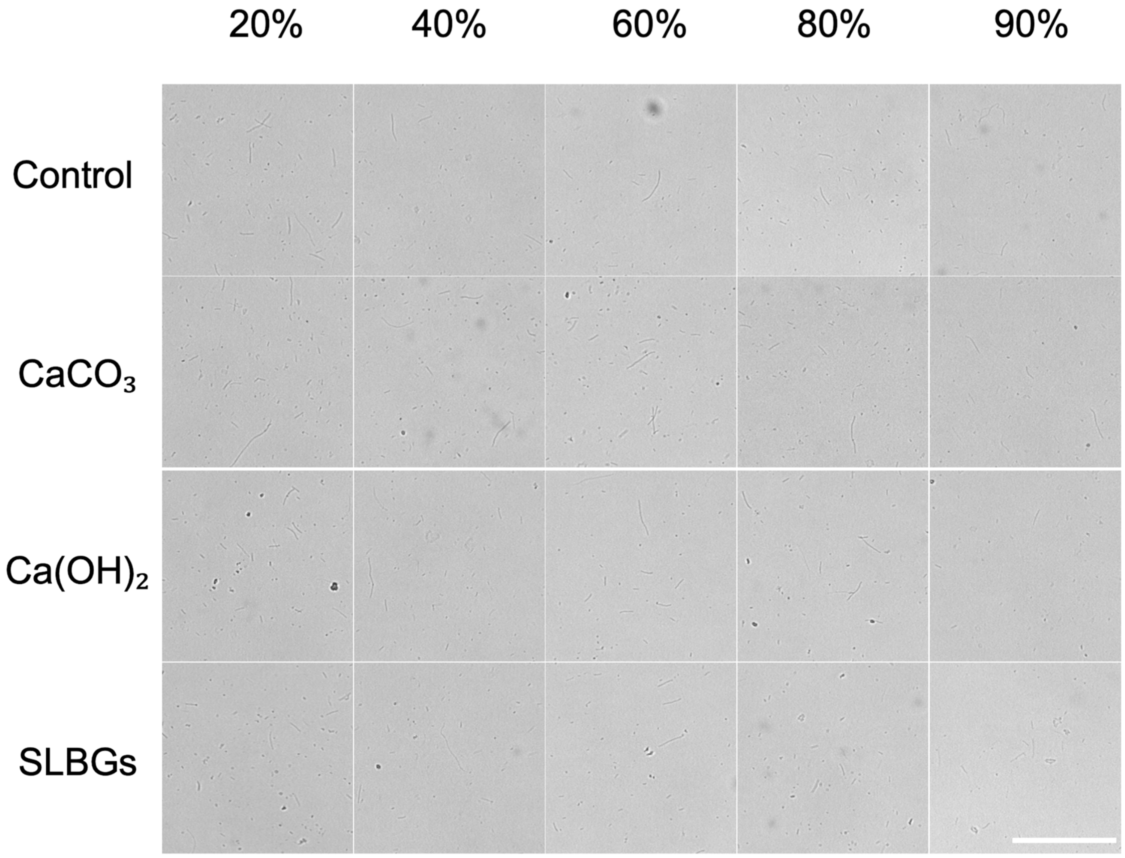

Supplementary Fig. 10. Bright-field images of *E. coli* treated with each disinfectant (control, $CaCO_3$, $Ca(OH)_2$, or SLBGs) under various humidity conditions for 3 h. The percentage (20%, 40%, 60%, 80%, or 90%) shown in the figure is the humidity set by the thermo-hygrostat. Images were captured using a fluorescence microscope equipped with a 20x objective. Bar, 100 μm.
